# Machine learning in the prediction of immunotherapy response and prognosis of melanoma: a systematic review and meta-analysis

**DOI:** 10.3389/fimmu.2024.1281940

**Published:** 2024-05-21

**Authors:** Juan Li, Kena Dan, Jun Ai

**Affiliations:** ^1^ Department of Dermatology, Chongqing Dangdai Plastic Surgery Hospital, Chongqing, China; ^2^ Department of Dermatology, The Third Affiliated Hospital of Chongqing Medical University, Chongqing, China; ^3^ Department of Dermatology, Chongqing Huamei Plastic Surgery Hospital, Chongqing, China

**Keywords:** machine learning, prediction, melanoma, immune checkpoint inhibitor, meta-analysis

## Abstract

**Background:**

The emergence of immunotherapy has changed the treatment modality for melanoma and prolonged the survival of many patients. However, a handful of patients remain unresponsive to immunotherapy and effective tools for early identification of this patient population are still lacking. Researchers have developed machine learning algorithms for predicting immunotherapy response in melanoma, but their predictive accuracy has been inconsistent. Therefore, the present systematic review and meta-analysis was performed to comprehensively evaluate the predictive accuracy of machine learning in melanoma response to immunotherapy.

**Methods:**

Relevant studies were searched in PubMed, Web of Sciences, Cochrane Library, and Embase from their inception to July 30, 2022. The risk of bias and applicability of the included studies were assessed using the Prediction Model Risk of Bias Assessment Tool (PROBAST). Meta-analysis was performed on R4.2.0.

**Results:**

A total of 36 studies consisting of 30 cohort studies and 6 case-control studies were included. These studies were mainly published between 2019 and 2022 and encompassed 75 models. The outcome measures of this study were progression-free survival (PFS), overall survival (OS), and treatment response. The pooled c-index was 0.728 (95%CI: 0.629–0.828) for PFS in the training set, 0.760 (95%CI: 0.728–0.792) and 0.819 (95%CI: 0.757–0.880) for treatment response in the training and validation sets, respectively, and 0.746 (95%CI: 0.721–0.771) and 0.700 (95%CI: 0.677–0.724) for OS in the training and validation sets, respectively.

**Conclusion:**

Machine learning has considerable predictive accuracy in melanoma immunotherapy response and prognosis, especially in the former. However, due to the lack of external validation and the scarcity of certain types of models, further studies are warranted.

## 1 Introduction

Melanoma is a potentially fatal and highly malignant melanocytic tumor with the highest metastasis and mortality rates among all skin cancers (1). Melanoma accounts for < 5% of all skin cancers but is responsible for 80% of skin cancer deaths. The incidence and mortality of melanoma continue to rise every year (2). Melanoma commonly develops in the skin but can also occur in the mucosa and organs. Confirmed risk factors for cutaneous melanoma include ultraviolet radiation and subsequent sunburn (3). For primary melanoma without lymph node metastases, the 5-year relative survival is 98% for stage I melanoma and 90% for stage II melanoma (4). However, melanoma often metastasizes to the lymph nodes first, and metastatic melanoma has a very poor prognosis. Melanomas that are identified early are generally removed by extensive local resection and staged by sentinel lymph node biopsy. Advanced melanoma, on the other hand, requires a multimodal approach consisting of systemic treatments such as targeted therapy (e.g., targeted BRAF inhibition (BRAFi)) and immune checkpoint inhibitors (ICIs) (5).

Immunotherapy exerts antitumor activities by enhancing immune responses in the body. However, hyperactivation of the immune system can damage normal cells. The advent of cytotoxic T-lymphocyte-associated protein 4 inhibitors (CTLA4 inhibitors) and programmed death ligand 1 inhibitors (PD-1 inhibitors) has completely revolutionized the management and treatment of various cancers and especially improved the systemic treatment of advanced melanoma (6). ICIs are superior to chemotherapy alone in the treatment of melanoma, but ICI combination therapy is even more effective than ICIs alone. As a result, nivolumab plus ipilimumab has been approved for the treatment of metastatic melanoma (7). A previous study showed that ipilimumab can increase the long-term survival rate (3 years or longer) of metastatic melanoma patients by about 20% (8). Although ICIs can improve the survival time of melanoma patients and maintain remission in responders, there are still a substantial number of patients who do not respond to ICIs. It was reported that less than 50% of patients respond to ICI monotherapy (9). Similarly, about 60%-70% of melanoma patients are unresponsive to anti-PD-1 monotherapy (nivolumab or pembrolizumab), and 40%-50% of patients are unresponsive to anti-CTLA-4 combination therapy (10). Therefore, early identification of the survival outcomes of melanoma patients receiving ICIs can aid in the development of rehabilitation and follow-up strategies and is of great significance in clinical practice. Unfortunately, there is currently a lack of such highly effective and accurate prediction tools.

With the rapid development of intelligent computing in recent years, machine learning has gained wide application in various fields and has been integrated into clinical practice. In particular, machine learning is commonly used for disease diagnosis and prognosis prediction (11). As machine learning continues to be improved and refined, challenges that were previously faced by researchers and clinicians are now easily resolved, and these models demonstrate great potentials in medical research, refinement of clinical care, and prediction of treatment and cancer prognosis. In fact, several studies have developed machine learning models for the early prediction of melanoma immunotherapy response and prognosis. However, these models were constructed using different methods and prediction variables such as clinical features, radiomics features, and a combination of both features. As a result, the predictive accuracy of these models has been inconsistent, which hinders the further development of artificial intelligence (AI) for the early prediction of immunotherapy response in melanoma. Here, we conducted a meta-analysis to systematically evaluate the predictive accuracy of machine learning in immunotherapy response and prognosis of melanoma in order to provide new insights into AI development in melanoma.

## 2 Materials and methods

Intervention studies involving animals or humans, and other studies that require ethical approval, must describe the authority that provided approval and the corresponding ethical approval code. This systematic review and meta-analysis was performed according to the Preferred Reporting Items for Systematic Reviews and Meta-Analyses (PRIMSA) guidelines (12).

### 2.1 Article search strategy

Relevant articles were systematically searched in PubMed, Cochrane Library, Embase, and Web of Science from their inception to July 30, 2022, without restrictions on country, language, or publication type. Search keywords included “Melanoma”, “machine learning”, and “Immune Checkpoint Inhibitors”. The complete search strategy for all databases is shown in **Supplementary Table 1**.

### 2.2 Inclusion and exclusion criteria

Inclusion criteria:

1. Patients who have been diagnosed with melanoma and received ICI treatment;
2. A complete machine learning predictive model for immunotherapy response or prognosis of melanoma;
3. Studies that only trained but did not validate the model using an independent validation set were included;
4. Different machine learning models based on the same dataset were included;
5. Studies published in English;
6. Randomized-controlled trials (RCTs), case-control studies, cohort studies, nested case-control studies, and case-cohort studies were included.

Exclusion criteria:

1. Studies that did not clearly describe ICI for the treatment of melanoma;
2. Only risk factors analysis was performed without the construction of a complete risk model;
3. Lack of metrics (receiver operating characteristic (ROC) curves, c-index, sensitivity, specificity, accuracy, recovery, precision, confusion matrix, diagnostic 2x2 table, F1 score, and calibration curve) that evaluate the accuracy of the machine learning model;
4. Meeting abstract without full text.

### 2.3 Article screening

The identified articles were imported into Endnote X20, and duplications were automatically and manually identified and removed. Titles and abstracts were screened, and the full texts of potentially eligible studies were downloaded for further review. Article screening was independently conducted and cross-checked by two researchers (Juan Li and Kena Dan). Any disagreement was resolved with the help of another researcher (Jun Ai).

### 2.4 Data extraction

A data extraction form was designed based on the modified Critical Appraisal and Data Extraction for Systematic Reviews of Prediction Modelling Studies (CHARMS) (13). Data that were extracted include:

1. Characteristics of included studies: Title, first author, year of publication, country, study type, and source of patients.
2. Characteristics of immunotherapy: Immunotherapy drug and duration of follow-up.
3. Characteristics of machine learning: Prediction goals, validation set, training set, method of variable screening/feature selection, model type, modeling variables, and model evaluation measures.

Data extraction was independently performed and cross-checked by two researchers (Juan Li and Kena Dan). Any disagreement was resolved with the help of another researcher (Jun Ai).

### 2.5 Risk of bias assessment

The risk of bias and applicability of the included studies were evaluated using the Prediction Model Risk of Bias Assessment Tool (PROBAST) (14). This tool includes 4 domains, namely participants, predictors, outcome, and analysis. The risk of bias in each included study was assessed as “high risk”, “low risk” or “unclear risk”. This process was independently completed by two researchers (Juan Li and Kena Dan), and any disagreement was resolved with the help of a third researcher (Jun Ai).

### 2.6 Outcome measures

The outcome measures for this systematic review were overall survival (OS), progression-free survival (PFS), and objective response rate (ORR) of ICI-treated melanoma patients.

### 2.7 Data analysis

A meta-analysis was performed on the c-index and accuracy of machine learning models. If the c-index lacks a 95% confidence interval (IC) and standard error, the standard error was estimated using the methods by Debray TP et al (15). Given the differences in variables and parameters used in various machine learning models, a random effects model was used for the meta-analysis of c-index. In addition, sensitivity and specificity were meta-analyzed using a bivariate mixed effects model. Subgroup analysis was performed based on each type of modeling variables (genomics, radiomics, and clinical characteristics. All meta-analyses were completed on R4.2.0 (R development Core Team, Vienna, http://www.R-project.org).

## 3 Results

### 3.1 Process of article screening

**Figure 1:**
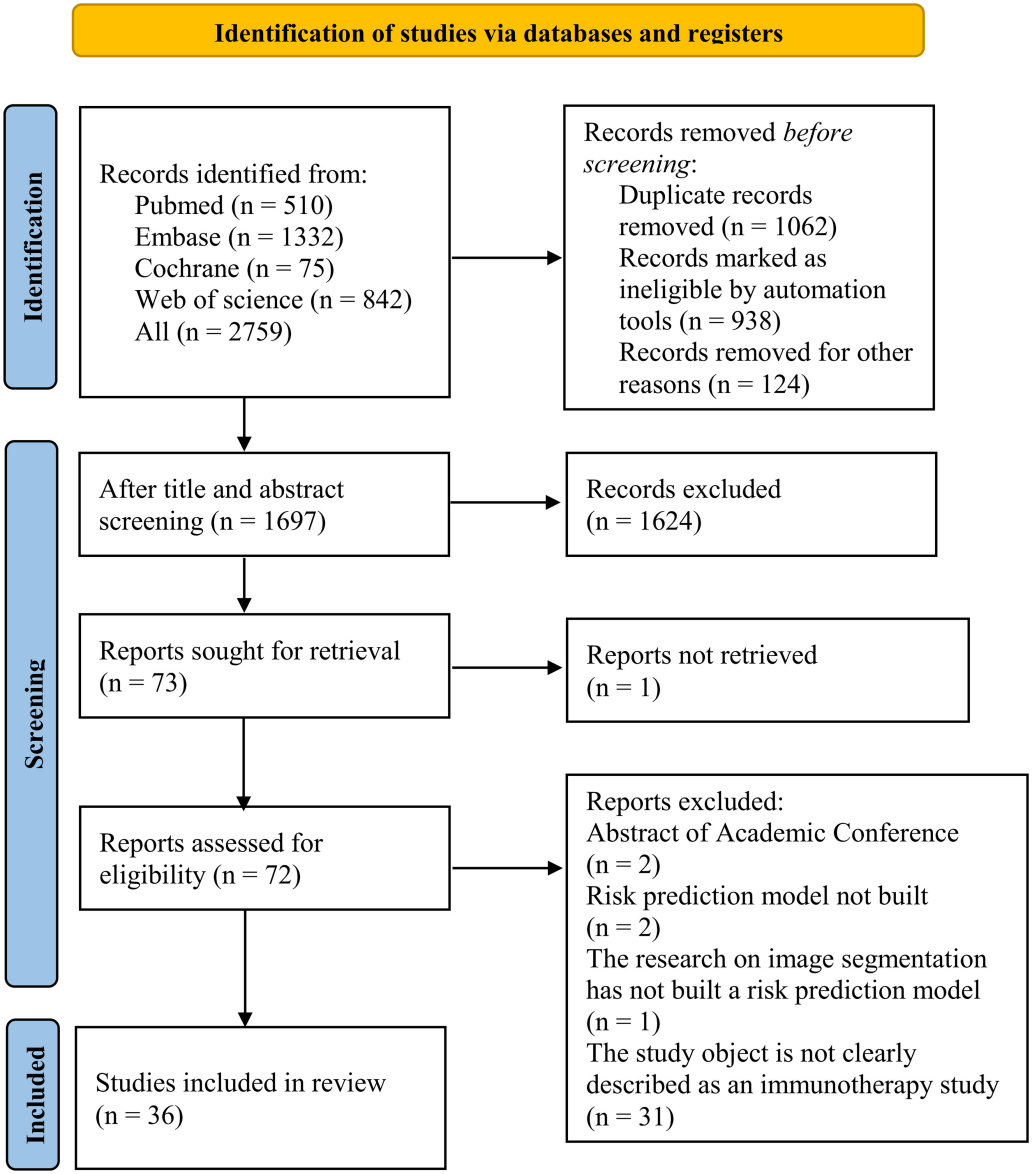
Process of study selection.

A total of 2,759 records were identified from the four databases, and 1,062 duplicate records were deleted. After title and abstract screening, 1,624 records were excluded, and the remaining 72 records were assessed for eligibility. After reviewing the full texts, 36 records were further excluded, and a final total of 36 records were included in the meta-analysis. The study selection process is shown in **Figure 1**.

### 3.2 General characteristics of included studies

The year of publication of the 36 included studies was primarily between 2019 and 2022. Of the 36 studies, 18 were conducted in China (16–33) and 5 were conducted in Germany (34–38). The primary outcome measures were treatment response, OS, and PFS. The included studies encompassed 75 models, including 45 OS prediction models, 21 treatment response models, and 6 PFS prediction models. In terms of modeling variables, there were 17 clinical features-based models, 27 radiomics-based risk models, and 31 genomics-based models. The specific characteristics of the included studies (16–50) are summarized in **Table 1**.

**Table 1:** General characteristics of included studies.

Author	Year	Country	Type	Patients	Immunotherapy	Object	Samples	Training set	Validation set	Feature selection	Model	Variables
Gang Hu (16)	2022	China	cohort	TCGA GTEx GEO	PD-1 PD-L1 CTLA4	OS	454	454	NA	Univariate + multivariate Cox	Cox	Genomics
Luyao Tian (17)	2022	China	cohort	GEO	PD-1	OS	87	87	NA	LASSO and random forest plot	Cox	Genomics
Kuixia Xie (18)	2022	China	cohort	TCGA-SKCM	PD-1	OS	322	68	144 110	Univariate + multivariate Cox	Cox	Genomics
Yalin Xie (19)	2022	China	cohort	TCGA	PD-1 CTLA-4	OS	471	471	NA	LASSO	Cox	Genomics
Rui Mao (20)	2021	China	cohort	TCGA	PD-1 CTLA-4	OS	454	210	231	Univariate + multivariate Cox	Cox	Genomics
Liuxing Wu (21)	2021	China	cohort	TCGA GEO	PD-1 CTLA-4	OS	1513	457	758 298	Univariate + multivariate Cox	Cox	Genomics
Ziqian Xu (22)	2021	China	cohort	TCGA FerrD	PD-1 CTLA-4	OS	457	457	NA	Univariate + multivariate Cox	Cox	Genomics
Xi Chen (23)	2021	China	cohort	Single center	PD-1 CTLA-4	TR	50	50	NA	LASSO	LR	Radiomics
Sitong Zhou (24)	2021	China	cohort	TCGA	PD-1 CTLA-4	OS	422	422	NA	LASSO	Cox	Genomics
Gang Li (25)	2021	China	cohort	TCGA	PD-1 CTLA-4	OS	513	453	60	Univariate + multivariate Cox	COX	Genomics
Yangyang Zeng (26)	2021	China	cohort	TCGA	PD-1 CTLA-4	OS	471	471	NA	LASSO	COX	Genomics
Xuan Wang (27)	2021	China	cohort	Single center	PD-1 CTLA-4	PFS	69	69	69	LASSO	COX	Genomics
Yang Sheng (28)	2020	China	cohort	TCGA	PD-1 CTLA-4	OS	381	381	NA	Univariate + multivariate Cox	COX	Genomics
Maolang Tian (29)	2020	China	cohort	TCGA GEO	PD-1 CTLA-4	OS	448	321	127	LASSO	COX	Genomics
Kai Kang (30)	2020	China	cohort	TCGA	PD-1 CTLA-4	OS	467	467	NA	Univariate + multivariate Cox	COX	Genomics
Zhi-long Wang (31)	2020	China	Case-control	Single center	PD-1 CTLA-4	TR	50	34	16	LASSO	LR	Radiomics
Xuanyi Wang (32)	2020	China	Case-control	GEO	PD-1	TR	79	79	NA	LASSO	RF	Genomics
Depei Li (33)	2020	China	cohort	Multicenter	PD-1 CTLA-4	OS	8078	3542	4536	Univariate + multivariate Cox	COX	Clinical characteristics
Katharina Filipski (34)	2021	Germany	Case-control	TCGA	PD-1	TR	396	396	396	LASSO	LR	Genomics
Amadeus Schraag (35)	2019	Germany	Case-control	Single center	PD-1 CTLA-4	OS TR	103	69	34	Univariate + multivariate COX	LR COX	Clinical characteristics
Andreas Stefan Brendlin (36)	2021	Germany	cohort	Single center	PD-1 CTLA-4	TR	140	70	70	Stepwise logistic regression	RF	Radiomics
Felix Peisen (37)	2022	Germany	cohort	CMMR	PD-L1 CTLA4	OS TR	262	262	NA	Not described	RF	Radiomics
Haidara Almansour (38)	2022	Germany	cohort	Single center	PD-1 CTLA-4	OS PFS	152	152	NA	Univariate + multivariate COX	Cox	Radiomics
Gabriela Marsavela (39)	2020	Australia.	cohort	Multicenter	PD-1 CTLA-4	PFS	110	110	NA	LASSO	COX	Genomics
Inês Pires da Silva (40)	2022	Australia	cohort	Multicenter	PD-1 CTLA-4	OS PFS	1511	500	419 592	LASSO	Cox	Clinical characteristics
Stefan Diem (41)	2015	UK	Case-control	Single center	CTLA-4	OS	134	134	NA	Univariate + multivariate COX	COX	Clinical characteristics
Karla A. Lee (9)	2022	UK	cohort	TCGA	PD-1 CTLA-4	ORR PFS	165	165	NA	LASSO	LASSO	Genomics
Anthime Flaus (42)	2022	France	cohort	Multicenter	PD-1	OS PFS	56	56	NA	LASSO	RF,ANN,NB,LR和SVM	Radiomics
Gulnur Ungan (43)	2022	France	cohort	Single center	PD-1	OS TR	71	71	NA	Boruta	LR	Radiomics
Gabriele Madonna (44)	2021	Italy	cohort	Single center	PD-1 CTLA-4	RFS OS	578	462	116	Univariate + multivariate COX	cox	Clinical characteristics
Alice Indini (45)	2019	Italy	Case-control	Single center	PD-1	PFS OS	173	173	NA	Univariate + multivariate COX	COX	Clinical characteristics
Lucas Basler (46)	2020	Switzerland	cohort	Single center	PD-1 CTLA-4	TR	112	112	NA	Univariate	LR	Clinical characteristics
Simon Burgermeister (47)	2022	Switzerland	cohort	Single center	PD-1 CTLA-4	OS	94	94	NA	Univariate + multivariate COX	Cox	Radiomics
Laurent Dercle (48)	2022	US	cohort	Vol-PACT	PD-1	OS TR	575	252	287	Not described	RF	Radiomics
Deirdre Kelly (49)	2021	Canada	cohort	Single center	PD-1 CTLA-4	TR	71	75	NA	Univariate + multivariate	LR	Clinical characteristics
Max J. Karlsson (50)	2021	Sweden	cohort	Single center	PD-1 CTLA-4	PFS	109	109	NA	LASSO	RF	Clinical characteristics

### 3.3 Risk of bias assessment

**Figure 2:**
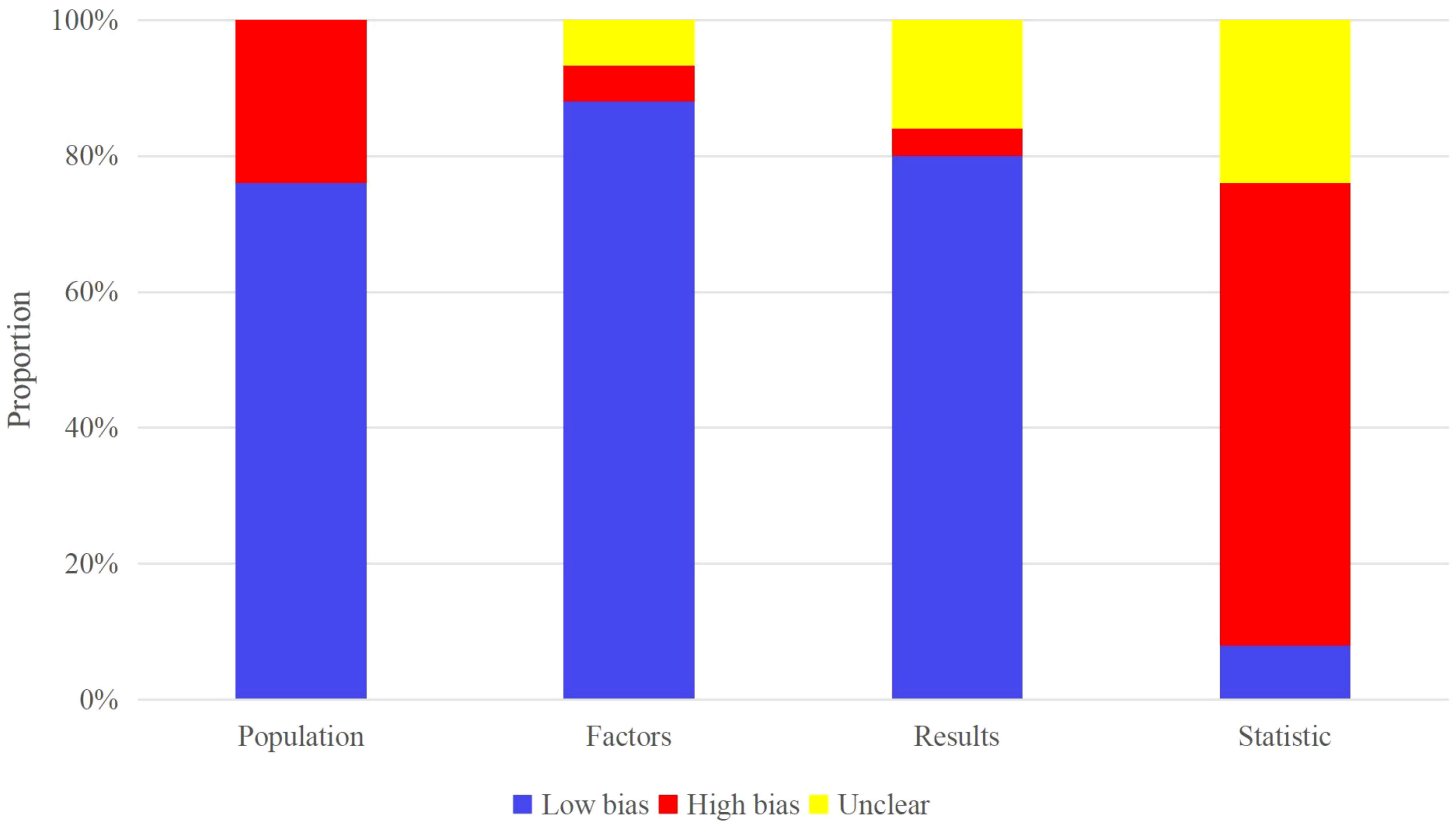
Risk of bias assessment.

All included studies evaluated the OS, treatment response, and PFS of melanoma patients after ICI treatment. Patients used for OS and PFS evaluation mainly originated from the respective cohort studies in registered databases and single-center electronic case databases. PROBAST assessment indicated that studies from the registered databases had a low risk of bias while those from the single-center electronic case database had a high risk of bias. Patients used for treatment response evaluation originated from case-control studies in registered databases or single-center electronic case databases. Most studies did not have >20 events per variable (EPV) or >100 patients in the independent validation set. Therefore, these two parameters constituted the primary sources of high bias in the included models (**Figure 2**).

### 3.4 Meta-analysis

The OS, treatment response, and PFS data were pooled from the 36 studies to determine the c-index (95% CI) of each outcome measure predicted by different models and modeling variables.

Genomics-based prediction models showed the best performance in OS prediction. The pooled c-index was 0.805 (95%CI: 0.731–0.879) in the training set and 0.694 (95%CI: 0.670–0.718) in the validation set, and the logistic regression model performed best with a c-index of 0.882 (95%CI: 0.835–0.929). Radiomics-based models ranked second in OS prediction (pooled c-index: 0.733 (95%CI: 0.722–0.745) in the training set), followed by clinical features-based models [pooled c-index: 0.719 (95%CI: 0.664–0.774) in the training set and 0.650 (95%CI: 0.641–0.659) in the validation set]. The overall c-index for OS was 0.746 (95%CI: 0.721–0.771) in the training set and 0.700 (95%CI: 0.677–0.724) in the validation set (**Table 2**).

**Table 2:** Meta-analysis of c-index of machine learning in OS prediction for ICI-treated melanoma patients.

Modeling variable	Model	Training set		Validation set	
		Number	c-index	Number	c-index
Geonomics					
	cox	2	0.810[0.593 ~ 1.027]	28	0.694[0.670 ~ 0.718]
	LR	3	0.882[0.835 ~ 0.929]	NA	NA
	RF	3	0.710[0.562 ~ 0.858]	NA	NA
	NB	1	0.820[0.724 ~ 0.916]	NA	NA
	SVM	1	0.820[0.724 ~ 0.916]	NA	NA
	ANN	1	0.840[0.749 ~ 0.931]	NA	NA
	Overall	11	0.805[0.731 ~ 0.879]	28	0.694[0.670 ~ 0.718]
Radomics					
	cox	18	0.739[0.725 ~ 0.752]	NA	NA
	LR	2	0.715[0.689 ~ 0.742]	NA	NA
	RF	3	0.719[0.668 ~ 0.769]	NA	NA
	Overall	23	0.733[0.722 ~ 0.745]	NA	NA
Clinical characteristics					
	cox	6	0.744[0.682 ~ 0.805]	2	0.650[0.641 ~ 0.659]
	RF	3	0.666[0.495 ~ 0.837]	NA	NA
	Overall	9	0.719[0.664 ~ 0.774]	2	0.650[0.641 ~ 0.659]
Overall		43	0.746[0.721 ~ 0.771]	34	0.700[0.677 ~ 0.724]

For the prediction of treatment response, the pooled c-index of radiomics-based models was 0.802 (95%CI: 0.739–0.866) in the training set and 0.813 (95%CI: 0.711–0.915) in the validation set. The pooled c-index of genomics-based models was 0.760 (95%CI: 0.668–0.853) in the training set and 0.857 (95%CI: 0.698–1.000) in the validation set. The pooled c-index of clinical characteristics-based models was 0.723 (95%CI: 0.699–0.747). The overall c-index for treatment response was 0.760 (95%CI: 0.728–0.792) in the training set and 0.819 (95%CI: 0.757–0.880) in the validation set (**Table 3**).

**Table 3:** Meta-analysis of c-index of machine learning in treatment response prediction for ICI-treated melanoma patients.

Modeling variable	Model	Training set		Validation set	
		Number	c-index	Number	c-index
Genomics					
	LR	4	0.723[0.659 ~ 0.786]	NA	NA
	RF	1	0.895[0.867 ~ 0.923]	NA	NA
	SVM	1	0.750[0.614 ~ 0.886]	1	0.857[0.698 ~ 1.000]
	Overall	6	0.760[0.668 ~ 0.853]	1	0.857[0.698 ~ 1.000]
Radiomics					
	LR	3	0.796[0.756 ~ 0.836]	2	0.813[0.711 ~ 0.915]
	RF	3	0.779[0.646 ~ 0.912]	1	0.710[0.508 ~ 0.912]
	SVM	2	0.871[0.794 ~ 0.948]	NA	NA
	overall	8	0.802[0.739 ~ 0.866]	3	0.813[0.711 ~ 0.915]
Clinical characteristics					
	LR	3	0.704[0.678 ~ 0.730]	NA	NA
	RF	3	0.738[0.711 ~ 0.765]	NA	NA
	overall	6	0.723[0.699 ~ 0.747]	NA	NA
Overall		20	0.760[0.728 ~ 0.792]	4	0.819[0.757 ~ 0.880]

Only genomics-based machine learning models were included for PFS prediction, but a validation set was lacking for the calculation of c-index. The overall c-index for PFS was 0.728 (95%CI: 0.629–0.828) in the training set (**Table 4**).

**Table 4:** Meta-analysis of c-index of machine learning in PFS prediction for ICI-treated melanoma patients.

Model	Number	c-index
cox	1	0.785[0.671 ~ 0.899]
LR	1	0.640[0.515 ~ 0.765]
RF	1	0.900[0.827 ~ 0.973]
NB	1	0.690[0.570 ~ 0.810]
SVM	1	0.630[0.504 ~ 0.756]
ANN	1	0.690[0.570 ~ 0.810]
Overall	6	0.728[0.629 ~ 0.828]

## 4 Discussion

### 4.1 Summary of the main findings

We performed a meta-analysis of the outcome measures of melanoma patients receiving ICI from 36 studies, and the pooled c-index showed that machine learning exhibits considerable predictive accuracy in melanoma immunotherapy response and prognosis. Our results showed that the genomics-based logistic regression model had the best performance in OS prediction [c-index 0.882 (95%CI: 0.835–0.929)], while the genomics-based random forest plot model performed best in treatment response and PS prediction [c-index 0.895 (95%CI: 0.867–0.923) and 0.900 (95%CI: 0.827–0.973), respectively]. Overall, we found that machine learning had the highest predictive accuracy for immunotherapy response in melanoma patients [0.760 (95%CI: 0.728–0.792) in the training set and 0.819 (95%CI: 0.757–0.880) in the validation set].

### 4.2 Comparison with previous reviews

Several studies have investigated the application of AI in the treatment and management of advanced melanoma. A review by Guerrisi et al. has discussed the application significance and challenges of AI in melanoma treatment but failed to assess the risk of bias and accuracy of existing machine learning models (51). Valenti and colleagues have provided an overview of the application of multi-omics (genomics, transcriptomics, proteomics, metabolomics and radiomics) in evaluating the immunotherapy response of melanoma. Many of these multi-omics models were highly dependent on machine learning, and their accuracy was not systematically described (52). Furthermore, another systematic review by Guerrisi et al. revealed that radiomics is a new robust approach for predicting targeted therapy and immunotherapy of melanoma (53). Though, none of these reviews has quantitatively assessed the predictive accuracy of the various machine learning models in the immunotherapy response and prognosis of melanoma. To fill in this gap, our systematic review has quantitatively determined the predictive accuracy of relevant radiomics-, genomics-, and clinical features-based machine learning models in order to provide valuable insights into subsequent development of AI tools.

### 4.3 Modeling variables

The selection of modeling variables is critical for machine learning. Common modeling variables for cancer prediction models are genomics, transcriptomics, proteomics, metabolomics, radiomics, and clinical features. In particular, clinical features often play an important role in model construction due to their ease of acquisition and good interpretability. Therefore, clinical features-based models are viewed as highly valuable by clinicians in predicting tumor treatment response or prognosis, especially in solid tumor prognosis. Moreover, machine learning models that integrate tumor stages have been shown to demonstrate superior predictive accuracy than other omics-based models. The emergence of ICIs has completely changed the treatment modality and prolonged the PFS and OS of melanoma patients (54). Despite the success of immunotherapy, treatment response and prognosis still vary greatly among patients, rendering many patients unable to benefit from these treatments. At present, PFS and OS predictions are still primarily based on clinical features. Our results showed that clinical features-, radiomics- and genomics-based machine learning models all exhibit favorable predictive accuracy in melanoma treatment response and prognosis. In particular, radiomics-based models have demonstrated promising application prospects in treatment response prediction in cancer patients, which is consistent with the findings by Zhang et al (55).

### 4.5 Limitations

There are several limitations to this study. First, due to practical reasons and the lack of external validation, the c-index of the training set was relatively high. Second, the low number of certain types of models in the included studies impeded the assessment of their predictive accuracy. Last, only a few of the included studies reported a diagnostic continency table or sensitivity and specificity for predicting treatment response, OS and PFS. Therefore, sensitivity and specificity will need to be further analyzed in subsequent studies.

### 4.6 Future prospects

(1) Various adverse events (AEs) have been reported in melanoma patients receiving ICIs. Hence, early prediction of ICI-related AEs has important clinical implications in treatment decision-making. However, very few published studies have investigated the predictive performance of machine learning in AEs. Therefore, the performance of machine learning models in predicting the risk of immunotherapy-related AEs in melanoma should be further examined in future work (2). The selection of modeling variables is critical for machine learning. We hope to identify more efficient and convenient predictors in our subsequent studies (3). Although our study showed that radiomics-based models have favorable predictive accuracy in immunotherapy response and prognosis of melanoma, radiomics faces many drawbacks such as over-configuration of imaging equipment, segmentation specificity of regions of interest, diversity in the modeling process, and lack of external validation. Thus, the development of a rational guideline for standardizing the implementation of radiomics research is warranted.

## 5 Conclusion

Machine learning models, especially those based on radiomics, have promising predictive accuracy in melanoma immunotherapy response and prognosis. Radiomics has gained wide interest in the research community in recent years, and hence standardization of radiomics research is critical for minimizing the risks of heterogeneity and bias.

## Data availability statement

The original contributions presented in the study are included in the article/**Supplementary material**. Further inquiries can be directed to the corresponding author.

## Author contributions

JL: Data curation, Formal analysis, Investigation, Methodology, Writing – original draft, Writing – review & editing. KD: Data curation, Formal analysis, Investigation, Methodology, Writing – original draft, Writing – review & editing. JA: Conceptualization, Formal analysis, Writing – original draft, Writing – review & editing.
