# Supplementary material for: Machine learning in the prediction of immunotherapy response and prognosis of melanoma: a systematic review and meta-analysis
Source: Front Immunol. 2024 May 21;15:1281940. doi: 10.3389/fimmu.2024.1281940 (PMC11148209; doi:10.3389/fimmu.2024.1281940)
Supplement: Supplementary file 1 [file Table_1.docx]

***Supplementary Material***

**Supplementary Table 1.** Search strategy.
Search date: July 30, 2022
Databases searched: Web of Science, PubMed, EMBASE, and Cochrane Library

1. **Search Strategy in Web of science**

| **Number** | **Query** | **Results** |
| --- | --- | --- |
| #1 | TS=(Melanoma) OR TS=(Melanomas) OR TS=(Malignant Melanoma) OR TS=(Malignant Melanomas) OR TS=(Melanoma, Malignant) OR TS=(Melanomas, Malignant) OR TS=(fortner melanoma) OR TS=(malignant melanomatosis) OR TS=(melanocarcinoma) OR TS=(melanomalignoma) OR TS=(naevi and melanomas) OR TS=(naevocarcinoma) OR TS=(nevi and melanomas) OR TS=(nevocarcinoma) OR TS=(nodular melanoma) OR TS=(pigmentary cancer) | [182,636](https://www.webofscience.com/wos/woscc/summary/0e9a1580-abab-4735-96c3-6d589be6f87a-40d969fc/relevance/1) |
| #2 | TS=(machine learning) OR TS=(Transfer Learning) OR TS=(Deep learning) OR TS=(Ensemble Learning) OR TS=(artificial intelligence) OR TS=(Prediction model) OR TS=(random forest) OR TS=(artificial neural network') OR TS=(ANN) OR TS=(convolutional neural network) OR TS=(Naive Bayes mode) OR TS=(Support vector machine) OR TS=(SVM) OR TS=(Gradient Boosting Machine) OR TS=(GBM) OR TS=(Nomogram) OR TS=(XGboost) OR TS=(Decision tree) OR TS=(External validation) OR TS=(Cox) OR TS=(Radiomics) OR TS=(radiomic) OR TS=(radiogenomic) OR TS=(radiomics-based) OR TS=(radiomic signature) | [1,826,990](https://www.webofscience.com/wos/woscc/summary/909bb5c4-09b0-4c7c-bdcf-e8390afed9a7-40d9e671/relevance/1) |
| #3 | TS=(Immune Checkpoint Inhibitors) OR TS=(Immunotherapy) OR TS=(Checkpoint Inhibitors, Immune) OR TS=(Immune Checkpoint Inhibitor) OR TS=(Checkpoint Inhibitor, Immune) OR TS=(Immune Checkpoint Blockers) OR TS=(Checkpoint Blockers, Immune) OR TS=(Immune Checkpoint Blockade) OR TS=(Checkpoint Blockade, Immune) OR TS=(Immune Checkpoint Inhibition) OR TS=(Checkpoint Inhibition, Immune) OR TS=(PD-L1 Inhibitors) OR TS=(PD L1 Inhibitors) OR TS=(PD-L1 Inhibitor) OR TS=(PD L1 Inhibitor) OR TS=(Programmed Death-Ligand 1 Inhibitors) OR TS=(Programmed Death Ligand 1 Inhibitors) OR TS=(PD-1-PD-L1 Blockade) OR TS=(Blockade, PD-1-PD-L1) OR TS=(PD 1 PD L1 Blockade) OR TS=(CTLA-4 Inhibitors) OR TS=(CTLA 4 Inhibitors) OR TS=(CTLA-4 Inhibitor) OR TS=(CTLA 4 Inhibitor) OR TS=(Cytotoxic T-Lymphocyte-Associated Protein 4 Inhibitors) OR TS=(Cytotoxic T Lymphocyte Associated Protein 4 Inhibitors) OR TS=(Cytotoxic T-Lymphocyte-Associated Protein 4 Inhibitor) OR TS=(Cytotoxic T Lymphocyte Associated Protein 4 Inhibitor) OR TS=(PD-1 Inhibitors) OR TS=(PD 1 Inhibitors) OR TS=(PD-1 Inhibitor) OR TS=(Inhibitor, PD-1) OR TS=(PD 1 Inhibitor) OR TS=(Programmed Cell Death Protein 1 Inhibitor) OR TS=(Programmed Cell Death Protein 1 Inhibitors) | [174,176](https://www.webofscience.com/wos/woscc/summary/e6da3fe0-b0ea-4ad1-bec3-8d76d8850284-40da3f5d/relevance/1) |
| #4 | #1 AND #2 AND #3 | [842](https://www.webofscience.com/wos/woscc/summary/3703fc36-7bb3-4430-8326-23b3f00dec20-40da483a/relevance/1) |

**842 Results**

1. **Search Strategy in PUBMED**

| **Search number** | **Query** | | **Search Details** | **Results** |
| --- | --- | --- | --- | --- |
| 11 | ((("Melanoma"[Mesh]) OR ((((((((((((((((Melanoma[Title/Abstract]) OR (Melanomas[Title/Abstract])) OR (Malignant Melanoma[Title/Abstract])) OR (Malignant Melanomas[Title/Abstract])) OR (Melanoma, Malignant[Title/Abstract])) OR (Melanomas, Malignant[Title/Abstract])) OR (fortner melanoma[Title/Abstract])) OR (malignant melanomatosis[Title/Abstract])) OR (melanocarcinoma[Title/Abstract])) OR (melanomalignoma[Title/Abstract])) OR ("naevi and melanomas"[Title/Abstract])) OR (naevocarcinoma[Title/Abstract])) OR ("nevi and melanomas"[Title/Abstract])) OR (nevocarcinoma[Title/Abstract])) OR (nodular melanoma[Title/Abstract])) OR (pigmentary cancer[Title/Abstract]))) AND (("Machine Learning"[Mesh]) OR (((((((((((((((((((((((((machine learning[Title/Abstract]) OR (Transfer Learning[Title/Abstract])) OR (Deep learning[Title/Abstract])) OR (Ensemble Learning[Title/Abstract])) OR (artificial intelligence[Title/Abstract])) OR (Prediction model[Title/Abstract])) OR (random forest[Title/Abstract])) OR (artificial neural network[Title/Abstract])) OR (ANN[Title/Abstract])) OR (convolutional neural network[Title/Abstract])) OR (Naive Bayes mode[Title/Abstract])) OR (Support vector machine[Title/Abstract])) OR (SVM[Title/Abstract])) OR (Gradient Boosting Machine[Title/Abstract])) OR (GBM[Title/Abstract])) OR (Nomogram[Title/Abstract])) OR (XGboost[Title/Abstract])) OR (Decision tree[Title/Abstract])) OR (External validation[Title/Abstract])) OR (Cox[Title/Abstract])) OR (Radiomics[Title/Abstract])) OR (radiomic[Title/Abstract])) OR (radiogenomic[Title/Abstract])) OR (radiomics-based[Title/Abstract])) OR (radiomic signature[Title/Abstract])))) AND (("Immune Checkpoint Inhibitors"[Mesh]) OR (((((((((((((((((((((((((((((((((((Immune Checkpoint Inhibitors[Title/Abstract]) OR (Immunotherapy[Title/Abstract])) OR (Checkpoint Inhibitors, Immune[Title/Abstract])) OR (Immune Checkpoint Inhibitor[Title/Abstract])) OR (Checkpoint Inhibitor, Immune[Title/Abstract])) OR (Immune Checkpoint Blockers[Title/Abstract])) OR (Checkpoint Blockers, Immune[Title/Abstract])) OR (Immune Checkpoint Blockade[Title/Abstract])) OR (Checkpoint Blockade, Immune[Title/Abstract])) OR (Immune Checkpoint Inhibition[Title/Abstract])) OR (Checkpoint Inhibition, Immune[Title/Abstract])) OR (PD-L1 Inhibitors[Title/Abstract])) OR (PD L1 Inhibitors[Title/Abstract])) OR (PD-L1 Inhibitor[Title/Abstract])) OR (PD L1 Inhibitor[Title/Abstract])) OR (Programmed Death-Ligand 1 Inhibitors[Title/Abstract])) OR (Programmed Death Ligand 1 Inhibitors[Title/Abstract])) OR (PD-1-PD-L1 Blockade[Title/Abstract])) OR (Blockade, PD-1-PD-L1[Title/Abstract])) OR (PD 1 PD L1 Blockade[Title/Abstract])) OR (CTLA-4 Inhibitors[Title/Abstract])) OR (CTLA 4 Inhibitors[Title/Abstract])) OR (CTLA-4 Inhibitor[Title/Abstract])) OR (CTLA 4 Inhibitor[Title/Abstract])) OR (Cytotoxic T-Lymphocyte-Associated Protein 4 Inhibitors[Title/Abstract])) OR (Cytotoxic T Lymphocyte Associated Protein 4 Inhibitors[Title/Abstract])) OR (Cytotoxic T-Lymphocyte-Associated Protein 4 Inhibitor[Title/Abstract])) OR (Cytotoxic T Lymphocyte Associated Protein 4 Inhibitor[Title/Abstract])) OR (PD-1 Inhibitors[Title/Abstract])) OR (PD 1 Inhibitors[Title/Abstract])) OR (PD-1 Inhibitor[Title/Abstract])) OR (Inhibitor, PD-1[Title/Abstract])) OR (PD 1 Inhibitor[Title/Abstract])) OR (Programmed Cell Death Protein 1 Inhibitor[Title/Abstract])) OR (Programmed Cell Death Protein 1 Inhibitors[Title/Abstract]))) | | ("Melanoma"[MeSH Terms] OR ("Melanoma"[Title/Abstract] OR "Melanomas"[Title/Abstract] OR "malignant melanoma"[Title/Abstract] OR "malignant melanomas"[Title/Abstract] OR "melanoma malignant"[Title/Abstract] OR "melanomas malignant"[Title/Abstract] OR (("fortner"[All Fields] OR "fortner s"[All Fields]) AND "Melanoma"[Title/Abstract]) OR "malignant melanomatosis"[Title/Abstract] OR "melanocarcinoma"[Title/Abstract] OR "melanomalignoma"[Title/Abstract] OR "naevi and melanomas"[Title/Abstract] OR "naevocarcinoma"[Title/Abstract] OR "nevi and melanomas"[Title/Abstract] OR "nevocarcinoma"[Title/Abstract] OR "nodular melanoma"[Title/Abstract] OR ("pigmentary"[All Fields] AND "cancer"[Title/Abstract]))) AND ("Machine Learning"[MeSH Terms] OR ("Machine Learning"[Title/Abstract] OR "transfer learning"[Title/Abstract] OR "deep learning"[Title/Abstract] OR "ensemble learning"[Title/Abstract] OR "artificial intelligence"[Title/Abstract] OR "prediction model"[Title/Abstract] OR "random forest"[Title/Abstract] OR "artificial neural network"[Title/Abstract] OR "ANN"[Title/Abstract] OR "convolutional neural network"[Title/Abstract] OR ((("naive"[All Fields] OR "naives"[All Fields]) AND ("baye s"[All Fields] OR "bayes"[All Fields] OR "bayes s"[All Fields])) AND "mode"[Title/Abstract]) OR "support vector machine"[Title/Abstract] OR "SVM"[Title/Abstract] OR "gradient boosting machine"[Title/Abstract] OR "GBM"[Title/Abstract] OR "Nomogram"[Title/Abstract] OR "XGboost"[Title/Abstract] OR "decision tree"[Title/Abstract] OR "external validation"[Title/Abstract] OR "Cox"[Title/Abstract] OR "Radiomics"[Title/Abstract] OR "radiomic"[Title/Abstract] OR "radiogenomic"[Title/Abstract] OR "radiomics-based"[Title/Abstract] OR "radiomic signature"[Title/Abstract])) AND ("Immune Checkpoint Inhibitors"[MeSH Terms] OR ("Immune Checkpoint Inhibitors"[Title/Abstract] OR "Immunotherapy"[Title/Abstract] OR "checkpoint inhibitors immune"[Title/Abstract] OR "immune checkpoint inhibitor"[Title/Abstract] OR "checkpoint inhibitor immune"[Title/Abstract] OR "immune checkpoint blockers"[Title/Abstract] OR "checkpoint blockers immune"[Title/Abstract] OR "immune checkpoint blockade"[Title/Abstract] OR "checkpoint blockade immune"[Title/Abstract] OR "immune checkpoint inhibition"[Title/Abstract] OR "checkpoint inhibition immune"[Title/Abstract] OR "pd l1 inhibitors"[Title/Abstract] OR "pd l1 inhibitors"[Title/Abstract] OR "pd l1 inhibitor"[Title/Abstract] OR "pd l1 inhibitor"[Title/Abstract] OR "programmed death ligand 1 inhibitors"[Title/Abstract] OR "programmed death ligand 1 inhibitors"[Title/Abstract] OR "pd 1 pd l1 blockade"[Title/Abstract] OR "blockade pd 1 pd l1"[Title/Abstract] OR "pd 1 pd l1 blockade"[Title/Abstract] OR "ctla 4 inhibitors"[Title/Abstract] OR "ctla 4 inhibitors"[Title/Abstract] OR "ctla 4 inhibitor"[Title/Abstract] OR "ctla 4 inhibitor"[Title/Abstract] OR "cytotoxic t lymphocyte associated protein 4 inhibitors"[Title/Abstract] OR "cytotoxic t lymphocyte associated protein 4 inhibitors"[Title/Abstract] OR "cytotoxic t lymphocyte associated protein 4 inhibitor"[Title/Abstract] OR "cytotoxic t lymphocyte associated protein 4 inhibitor"[Title/Abstract] OR "pd 1 inhibitors"[Title/Abstract] OR "pd 1 inhibitors"[Title/Abstract] OR "pd 1 inhibitor"[Title/Abstract] OR "inhibitor pd 1"[Title/Abstract] OR "pd 1 inhibitor"[Title/Abstract] OR "programmed cell death protein 1 inhibitor"[Title/Abstract] OR "programmed cell death protein 1 inhibitors"[Title/Abstract])) | 510 |
| 10 | ("Immune Checkpoint Inhibitors"[Mesh]) OR (((((((((((((((((((((((((((((((((((Immune Checkpoint Inhibitors[Title/Abstract]) OR (Immunotherapy[Title/Abstract])) OR (Checkpoint Inhibitors, Immune[Title/Abstract])) OR (Immune Checkpoint Inhibitor[Title/Abstract])) OR (Checkpoint Inhibitor, Immune[Title/Abstract])) OR (Immune Checkpoint Blockers[Title/Abstract])) OR (Checkpoint Blockers, Immune[Title/Abstract])) OR (Immune Checkpoint Blockade[Title/Abstract])) OR (Checkpoint Blockade, Immune[Title/Abstract])) OR (Immune Checkpoint Inhibition[Title/Abstract])) OR (Checkpoint Inhibition, Immune[Title/Abstract])) OR (PD-L1 Inhibitors[Title/Abstract])) OR (PD L1 Inhibitors[Title/Abstract])) OR (PD-L1 Inhibitor[Title/Abstract])) OR (PD L1 Inhibitor[Title/Abstract])) OR (Programmed Death-Ligand 1 Inhibitors[Title/Abstract])) OR (Programmed Death Ligand 1 Inhibitors[Title/Abstract])) OR (PD-1-PD-L1 Blockade[Title/Abstract])) OR (Blockade, PD-1-PD-L1[Title/Abstract])) OR (PD 1 PD L1 Blockade[Title/Abstract])) OR (CTLA-4 Inhibitors[Title/Abstract])) OR (CTLA 4 Inhibitors[Title/Abstract])) OR (CTLA-4 Inhibitor[Title/Abstract])) OR (CTLA 4 Inhibitor[Title/Abstract])) OR (Cytotoxic T-Lymphocyte-Associated Protein 4 Inhibitors[Title/Abstract])) OR (Cytotoxic T Lymphocyte Associated Protein 4 Inhibitors[Title/Abstract])) OR (Cytotoxic T-Lymphocyte-Associated Protein 4 Inhibitor[Title/Abstract])) OR (Cytotoxic T Lymphocyte Associated Protein 4 Inhibitor[Title/Abstract])) OR (PD-1 Inhibitors[Title/Abstract])) OR (PD 1 Inhibitors[Title/Abstract])) OR (PD-1 Inhibitor[Title/Abstract])) OR (Inhibitor, PD-1[Title/Abstract])) OR (PD 1 Inhibitor[Title/Abstract])) OR (Programmed Cell Death Protein 1 Inhibitor[Title/Abstract])) OR (Programmed Cell Death Protein 1 Inhibitors[Title/Abstract])) | | "Immune Checkpoint Inhibitors"[MeSH Terms] OR "Immune Checkpoint Inhibitors"[Title/Abstract] OR "Immunotherapy"[Title/Abstract] OR "checkpoint inhibitors immune"[Title/Abstract] OR "immune checkpoint inhibitor"[Title/Abstract] OR "checkpoint inhibitor immune"[Title/Abstract] OR "immune checkpoint blockers"[Title/Abstract] OR "checkpoint blockers immune"[Title/Abstract] OR "immune checkpoint blockade"[Title/Abstract] OR "checkpoint blockade immune"[Title/Abstract] OR "immune checkpoint inhibition"[Title/Abstract] OR "checkpoint inhibition immune"[Title/Abstract] OR "pd l1 inhibitors"[Title/Abstract] OR "pd l1 inhibitors"[Title/Abstract] OR "pd l1 inhibitor"[Title/Abstract] OR "pd l1 inhibitor"[Title/Abstract] OR "programmed death ligand 1 inhibitors"[Title/Abstract] OR "programmed death ligand 1 inhibitors"[Title/Abstract] OR "pd 1 pd l1 blockade"[Title/Abstract] OR "blockade pd 1 pd l1"[Title/Abstract] OR "pd 1 pd l1 blockade"[Title/Abstract] OR "ctla 4 inhibitors"[Title/Abstract] OR "ctla 4 inhibitors"[Title/Abstract] OR "ctla 4 inhibitor"[Title/Abstract] OR "ctla 4 inhibitor"[Title/Abstract] OR "cytotoxic t lymphocyte associated protein 4 inhibitors"[Title/Abstract] OR "cytotoxic t lymphocyte associated protein 4 inhibitors"[Title/Abstract] OR "cytotoxic t lymphocyte associated protein 4 inhibitor"[Title/Abstract] OR "cytotoxic t lymphocyte associated protein 4 inhibitor"[Title/Abstract] OR "pd 1 inhibitors"[Title/Abstract] OR "pd 1 inhibitors"[Title/Abstract] OR "pd 1 inhibitor"[Title/Abstract] OR "inhibitor pd 1"[Title/Abstract] OR "pd 1 inhibitor"[Title/Abstract] OR "programmed cell death protein 1 inhibitor"[Title/Abstract] OR "programmed cell death protein 1 inhibitors"[Title/Abstract] | 119,699 |
| 9 | ((((((((((((((((((((((((((((((((((Immune Checkpoint Inhibitors[Title/Abstract]) OR (Immunotherapy[Title/Abstract])) OR (Checkpoint Inhibitors, Immune[Title/Abstract])) OR (Immune Checkpoint Inhibitor[Title/Abstract])) OR (Checkpoint Inhibitor, Immune[Title/Abstract])) OR (Immune Checkpoint Blockers[Title/Abstract])) OR (Checkpoint Blockers, Immune[Title/Abstract])) OR (Immune Checkpoint Blockade[Title/Abstract])) OR (Checkpoint Blockade, Immune[Title/Abstract])) OR (Immune Checkpoint Inhibition[Title/Abstract])) OR (Checkpoint Inhibition, Immune[Title/Abstract])) OR (PD-L1 Inhibitors[Title/Abstract])) OR (PD L1 Inhibitors[Title/Abstract])) OR (PD-L1 Inhibitor[Title/Abstract])) OR (PD L1 Inhibitor[Title/Abstract])) OR (Programmed Death-Ligand 1 Inhibitors[Title/Abstract])) OR (Programmed Death Ligand 1 Inhibitors[Title/Abstract])) OR (PD-1-PD-L1 Blockade[Title/Abstract])) OR (Blockade, PD-1-PD-L1[Title/Abstract])) OR (PD 1 PD L1 Blockade[Title/Abstract])) OR (CTLA-4 Inhibitors[Title/Abstract])) OR (CTLA 4 Inhibitors[Title/Abstract])) OR (CTLA-4 Inhibitor[Title/Abstract])) OR (CTLA 4 Inhibitor[Title/Abstract])) OR (Cytotoxic T-Lymphocyte-Associated Protein 4 Inhibitors[Title/Abstract])) OR (Cytotoxic T Lymphocyte Associated Protein 4 Inhibitors[Title/Abstract])) OR (Cytotoxic T-Lymphocyte-Associated Protein 4 Inhibitor[Title/Abstract])) OR (Cytotoxic T Lymphocyte Associated Protein 4 Inhibitor[Title/Abstract])) OR (PD-1 Inhibitors[Title/Abstract])) OR (PD 1 Inhibitors[Title/Abstract])) OR (PD-1 Inhibitor[Title/Abstract])) OR (Inhibitor, PD-1[Title/Abstract])) OR (PD 1 Inhibitor[Title/Abstract])) OR (Programmed Cell Death Protein 1 Inhibitor[Title/Abstract])) OR (Programmed Cell Death Protein 1 Inhibitors[Title/Abstract]) | | "immune checkpoint inhibitors"[Title/Abstract] OR "Immunotherapy"[Title/Abstract] OR "checkpoint inhibitors immune"[Title/Abstract] OR "immune checkpoint inhibitor"[Title/Abstract] OR "checkpoint inhibitor immune"[Title/Abstract] OR "immune checkpoint blockers"[Title/Abstract] OR "checkpoint blockers immune"[Title/Abstract] OR "immune checkpoint blockade"[Title/Abstract] OR "checkpoint blockade immune"[Title/Abstract] OR "immune checkpoint inhibition"[Title/Abstract] OR "checkpoint inhibition immune"[Title/Abstract] OR "pd l1 inhibitors"[Title/Abstract] OR "pd l1 inhibitors"[Title/Abstract] OR "pd l1 inhibitor"[Title/Abstract] OR "pd l1 inhibitor"[Title/Abstract] OR "programmed death ligand 1 inhibitors"[Title/Abstract] OR "programmed death ligand 1 inhibitors"[Title/Abstract] OR "pd 1 pd l1 blockade"[Title/Abstract] OR "blockade pd 1 pd l1"[Title/Abstract] OR "pd 1 pd l1 blockade"[Title/Abstract] OR "ctla 4 inhibitors"[Title/Abstract] OR "ctla 4 inhibitors"[Title/Abstract] OR "ctla 4 inhibitor"[Title/Abstract] OR "ctla 4 inhibitor"[Title/Abstract] OR "cytotoxic t lymphocyte associated protein 4 inhibitors"[Title/Abstract] OR "cytotoxic t lymphocyte associated protein 4 inhibitors"[Title/Abstract] OR "cytotoxic t lymphocyte associated protein 4 inhibitor"[Title/Abstract] OR "cytotoxic t lymphocyte associated protein 4 inhibitor"[Title/Abstract] OR "pd 1 inhibitors"[Title/Abstract] OR "pd 1 inhibitors"[Title/Abstract] OR "pd 1 inhibitor"[Title/Abstract] OR "inhibitor pd 1"[Title/Abstract] OR "pd 1 inhibitor"[Title/Abstract] OR "programmed cell death protein 1 inhibitor"[Title/Abstract] OR "programmed cell death protein 1 inhibitors"[Title/Abstract] | 119,026 |
| 8 | "Immune Checkpoint Inhibitors"[Mesh] | Most Recent | "Immune Checkpoint Inhibitors"[MeSH Terms] | 6,104 |
| 7 | ("Machine Learning"[Mesh]) OR (((((((((((((((((((((((((machine learning[Title/Abstract]) OR (Transfer Learning[Title/Abstract])) OR (Deep learning[Title/Abstract])) OR (Ensemble Learning[Title/Abstract])) OR (artificial intelligence[Title/Abstract])) OR (Prediction model[Title/Abstract])) OR (random forest[Title/Abstract])) OR (artificial neural network[Title/Abstract])) OR (ANN[Title/Abstract])) OR (convolutional neural network[Title/Abstract])) OR (Naive Bayes mode[Title/Abstract])) OR (Support vector machine[Title/Abstract])) OR (SVM[Title/Abstract])) OR (Gradient Boosting Machine[Title/Abstract])) OR (GBM[Title/Abstract])) OR (Nomogram[Title/Abstract])) OR (XGboost[Title/Abstract])) OR (Decision tree[Title/Abstract])) OR (External validation[Title/Abstract])) OR (Cox[Title/Abstract])) OR (Radiomics[Title/Abstract])) OR (radiomic[Title/Abstract])) OR (radiogenomic[Title/Abstract])) OR (radiomics-based[Title/Abstract])) OR (radiomic signature[Title/Abstract])) | | "Machine Learning"[MeSH Terms] OR ("Machine Learning"[Title/Abstract] OR "transfer learning"[Title/Abstract] OR "deep learning"[Title/Abstract] OR "ensemble learning"[Title/Abstract] OR "artificial intelligence"[Title/Abstract] OR "prediction model"[Title/Abstract] OR "random forest"[Title/Abstract] OR "artificial neural network"[Title/Abstract] OR "ANN"[Title/Abstract] OR "convolutional neural network"[Title/Abstract] OR ((("naive"[All Fields] OR "naives"[All Fields]) AND ("baye s"[All Fields] OR "bayes"[All Fields] OR "bayes s"[All Fields])) AND "mode"[Title/Abstract]) OR "support vector machine"[Title/Abstract] OR "SVM"[Title/Abstract] OR "gradient boosting machine"[Title/Abstract] OR "GBM"[Title/Abstract] OR "Nomogram"[Title/Abstract] OR "XGboost"[Title/Abstract] OR "decision tree"[Title/Abstract] OR "external validation"[Title/Abstract] OR "Cox"[Title/Abstract] OR "Radiomics"[Title/Abstract] OR "radiomic"[Title/Abstract] OR "radiogenomic"[Title/Abstract] OR "radiomics-based"[Title/Abstract] OR "radiomic signature"[Title/Abstract]) | 414,998 |
| 6 | ((((((((((((((((((((((((machine learning[Title/Abstract]) OR (Transfer Learning[Title/Abstract])) OR (Deep learning[Title/Abstract])) OR (Ensemble Learning[Title/Abstract])) OR (artificial intelligence[Title/Abstract])) OR (Prediction model[Title/Abstract])) OR (random forest[Title/Abstract])) OR (artificial neural network[Title/Abstract])) OR (ANN[Title/Abstract])) OR (convolutional neural network[Title/Abstract])) OR (Naive Bayes mode[Title/Abstract])) OR (Support vector machine[Title/Abstract])) OR (SVM[Title/Abstract])) OR (Gradient Boosting Machine[Title/Abstract])) OR (GBM[Title/Abstract])) OR (Nomogram[Title/Abstract])) OR (XGboost[Title/Abstract])) OR (Decision tree[Title/Abstract])) OR (External validation[Title/Abstract])) OR (Cox[Title/Abstract])) OR (Radiomics[Title/Abstract])) OR (radiomic[Title/Abstract])) OR (radiogenomic[Title/Abstract])) OR (radiomics-based[Title/Abstract])) OR (radiomic signature[Title/Abstract]) | | "machine learning"[Title/Abstract] OR "transfer learning"[Title/Abstract] OR "deep learning"[Title/Abstract] OR "ensemble learning"[Title/Abstract] OR "artificial intelligence"[Title/Abstract] OR "prediction model"[Title/Abstract] OR "random forest"[Title/Abstract] OR "artificial neural network"[Title/Abstract] OR "ANN"[Title/Abstract] OR "convolutional neural network"[Title/Abstract] OR ((("naive"[All Fields] OR "naives"[All Fields]) AND ("baye s"[All Fields] OR "bayes"[All Fields] OR "bayes s"[All Fields])) AND "mode"[Title/Abstract]) OR "support vector machine"[Title/Abstract] OR "SVM"[Title/Abstract] OR "gradient boosting machine"[Title/Abstract] OR "GBM"[Title/Abstract] OR "Nomogram"[Title/Abstract] OR "XGboost"[Title/Abstract] OR "decision tree"[Title/Abstract] OR "external validation"[Title/Abstract] OR "Cox"[Title/Abstract] OR "Radiomics"[Title/Abstract] OR "radiomic"[Title/Abstract] OR "radiogenomic"[Title/Abstract] OR "radiomics-based"[Title/Abstract] OR "radiomic signature"[Title/Abstract] | 409,354 |
| 5 | "Machine Learning"[Mesh] | Most Recent | "Machine Learning"[MeSH Terms] | 46,087 |
| 4 | ("Melanoma"[Mesh]) OR ((((((((((((((((Melanoma[Title/Abstract]) OR (Melanomas[Title/Abstract])) OR (Malignant Melanoma[Title/Abstract])) OR (Malignant Melanomas[Title/Abstract])) OR (Melanoma, Malignant[Title/Abstract])) OR (Melanomas, Malignant[Title/Abstract])) OR (fortner melanoma[Title/Abstract])) OR (malignant melanomatosis[Title/Abstract])) OR (melanocarcinoma[Title/Abstract])) OR (melanomalignoma[Title/Abstract])) OR ("naevi and melanomas"[Title/Abstract])) OR (naevocarcinoma[Title/Abstract])) OR ("nevi and melanomas"[Title/Abstract])) OR (nevocarcinoma[Title/Abstract])) OR (nodular melanoma[Title/Abstract])) OR (pigmentary cancer[Title/Abstract])) | | "Melanoma"[MeSH Terms] OR ("Melanoma"[Title/Abstract] OR "Melanomas"[Title/Abstract] OR "malignant melanoma"[Title/Abstract] OR "malignant melanomas"[Title/Abstract] OR "melanoma malignant"[Title/Abstract] OR "melanomas malignant"[Title/Abstract] OR (("fortner"[All Fields] OR "fortner s"[All Fields]) AND "Melanoma"[Title/Abstract]) OR "malignant melanomatosis"[Title/Abstract] OR "melanocarcinoma"[Title/Abstract] OR "melanomalignoma"[Title/Abstract] OR "naevi and melanomas"[Title/Abstract] OR "naevocarcinoma"[Title/Abstract] OR "nevi and melanomas"[Title/Abstract] OR "nevocarcinoma"[Title/Abstract] OR "nodular melanoma"[Title/Abstract] OR ("pigmentary"[All Fields] AND "cancer"[Title/Abstract])) | 148,921 |
| 3 | (((((((((((((((Melanoma[Title/Abstract]) OR (Melanomas[Title/Abstract])) OR (Malignant Melanoma[Title/Abstract])) OR (Malignant Melanomas[Title/Abstract])) OR (Melanoma, Malignant[Title/Abstract])) OR (Melanomas, Malignant[Title/Abstract])) OR (fortner melanoma[Title/Abstract])) OR (malignant melanomatosis[Title/Abstract])) OR (melanocarcinoma[Title/Abstract])) OR (melanomalignoma[Title/Abstract])) OR ("naevi and melanomas"[Title/Abstract])) OR (naevocarcinoma[Title/Abstract])) OR ("nevi and melanomas"[Title/Abstract])) OR (nevocarcinoma[Title/Abstract])) OR (nodular melanoma[Title/Abstract])) OR (pigmentary cancer[Title/Abstract]) | | "Melanoma"[Title/Abstract] OR "Melanomas"[Title/Abstract] OR "malignant melanoma"[Title/Abstract] OR "malignant melanomas"[Title/Abstract] OR "melanoma malignant"[Title/Abstract] OR "melanomas malignant"[Title/Abstract] OR (("fortner"[All Fields] OR "fortner s"[All Fields]) AND "Melanoma"[Title/Abstract]) OR "malignant melanomatosis"[Title/Abstract] OR "melanocarcinoma"[Title/Abstract] OR "melanomalignoma"[Title/Abstract] OR "naevi and melanomas"[Title/Abstract] OR "naevocarcinoma"[Title/Abstract] OR "nevi and melanomas"[Title/Abstract] OR "nevocarcinoma"[Title/Abstract] OR "nodular melanoma"[Title/Abstract] OR ("pigmentary"[All Fields] AND "cancer"[Title/Abstract]) | 131,856 |
| 2 | "Melanoma"[Mesh] | Most Recent | "Melanoma"[MeSH Terms] | 104,931 |

**510 Results**

1. **Search Strategy in Embase**

| **No.** | **Query** | **Results** |
| --- | --- | --- |
| #7 | #2 AND #4 AND #6 | 1332 |
| #6 | 'immune checkpoint inhibitors'/exp OR 'immune checkpoint inhibitors' OR immunotherapy:ab,ti OR 'checkpoint inhibitors, immune':ab,ti OR 'immune checkpoint inhibitor':ab,ti OR 'checkpoint inhibitor, immune':ab,ti OR 'immune checkpoint blockers':ab,ti OR 'checkpoint blockers, immune':ab,ti OR 'immune checkpoint blockade':ab,ti OR 'checkpoint blockade, immune':ab,ti OR 'immune checkpoint inhibition':ab,ti OR 'checkpoint inhibition, immune':ab,ti OR 'pd-l1 inhibitors':ab,ti OR 'pd l1 inhibitors':ab,ti OR 'pd-l1 inhibitor':ab,ti OR 'pd l1 inhibitor':ab,ti OR 'programmed death-ligand 1 inhibitors':ab,ti OR 'programmed death ligand 1 inhibitors':ab,ti OR 'pd-1-pd-l1 blockade':ab,ti OR 'blockade, pd-1-pd-l1':ab,ti OR 'pd 1 pd l1 blockade':ab,ti OR 'ctla-4 inhibitors':ab,ti OR 'ctla 4 inhibitors':ab,ti OR 'ctla-4 inhibitor':ab,ti OR 'ctla 4 inhibitor':ab,ti OR 'cytotoxic t-lymphocyte-associated protein 4 inhibitors':ab,ti OR 'cytotoxic t lymphocyte associated protein 4 inhibitors':ab,ti OR 'cytotoxic t-lymphocyte-associated protein 4 inhibitor':ab,ti OR 'cytotoxic t lymphocyte associated protein 4 inhibitor':ab,ti OR 'pd-1 inhibitors':ab,ti OR 'pd 1 inhibitors':ab,ti OR 'pd-1 inhibitor':ab,ti OR 'inhibitor, pd-1':ab,ti OR 'pd 1 inhibitor':ab,ti OR 'programmed cell death protein 1 inhibitor':ab,ti OR 'programmed cell death protein 1 inhibitors':ab,ti | 173255 |
| #5 | 'immune checkpoint inhibitor'/exp | 12990 |
| #4 | 'machine learning'/exp OR 'machine learning' OR 'transfer of learning':ab,ti OR 'deep learning':ab,ti OR 'ensemble learning':ab,ti OR 'artificial intelligence':ab,ti OR 'prediction model':ab,ti OR 'random forest':ab,ti OR 'artificial neural network':ab,ti OR ann:ab,ti OR 'convolutional neural network':ab,ti OR 'naive bayes mode':ab,ti OR 'support vector machine':ab,ti OR svm:ab,ti OR 'gradient boosting machine':ab,ti OR gbm:ab,ti OR nomogram:ab,ti OR xgboost:ab,ti OR 'decision tree':ab,ti OR 'external validation':ab,ti OR cox:ab,ti OR radiomics:ab,ti OR radiomic:ab,ti OR radiogenomic:ab,ti OR 'radiomics based':ab,ti OR 'radiomic signature':ab,ti | 821,881 |
| #3 | 'machine learning'/exp | 316633 |
| #2 | 'melanoma'/exp OR melanoma OR melanomas:ab,ti OR 'malignant melanoma':ab,ti OR 'malignant melanomas':ab,ti OR 'melanoma, malignant':ab,ti OR 'melanomas, malignant':ab,ti OR 'fortner melanoma':ab,ti OR 'malignant melanomatosis':ab,ti OR melanocarcinoma:ab,ti OR melanomalignoma:ab,ti OR (naevi:ab,ti AND melanomas:ab,ti) OR naevocarcinoma:ab,ti OR (nevi:ab,ti AND melanomas:ab,ti) OR nevocarcinoma:ab,ti OR 'nodular melanoma':ab,ti OR 'pigmentary cancer':ab,ti | 242416 |
| #1 | 'melanoma'/exp | 188868 |

**1332 Results**

1. **Search Strategy in the Cochrane Library**

| **ID** | **Search** | **Results** |
| --- | --- | --- |
| #1 | MeSH descriptor: [Melanoma] explode all trees | 1969 |
| #2 | (Melanoma):ti,ab,kw OR (Melanomas):ti,ab,kw OR (Malignant Melanoma):ti,ab,kw OR (Malignant Melanomas):ti,ab,kw OR (Melanoma, Malignant):ti,ab,kw | 6026 |
| #3 | (Melanomas, Malignant):ti,ab,kw OR (fortner melanoma):ti,ab,kw OR (malignant melanomatosis):ti,ab,kw OR (melanocarcinoma):ti,ab,kw OR (melanomalignoma):ti,ab,kw | 87 |
| #4 | (naevi and melanomas):ti,ab,kw OR (naevocarcinoma):ti,ab,kw OR (nevi and melanomas):ti,ab,kw OR (nevocarcinoma):ti,ab,kw OR (nodular melanoma):ti,ab,kw | 44 |
| #5 | (pigmentary cancer):ti,ab,kw | 9 |
| #6 | #1 OR #2 OR #3 OR #4 OR #5 | 6035 |
| #7 | MeSH descriptor: [Machine Learning] explode all trees | 234 |
| #8 | (machine learning):ti,ab,kw OR (Transfer Learning):ti,ab,kw OR (Deep learning):ti,ab,kw OR (Ensemble Learning):ti,ab,kw OR (artificial intelligence):ti,ab,kw | 4772 |
| #9 | (Prediction model):ti,ab,kw OR (random forest):ti,ab,kw OR (artificial neural network):ti,ab,kw OR (ANN):ti,ab,kw OR (convolutional neural network):ti,ab,kw | 7554 |
| #10 | (Naive Bayes mode):ti,ab,kw OR (Support vector machine):ti,ab,kw OR (SVM):ti,ab,kw OR (Gradient Boosting Machine):ti,ab,kw OR (GBM):ti,ab,kw | 1440 |
| #11 | (Nomogram):ti,ab,kw OR (XGboost):ti,ab,kw OR (Decision tree):ti,ab,kw OR (External validation):ti,ab,kw OR (Cox):ti,ab,kw | 21486 |
| #12 | (Radiomics):ti,ab,kw OR (radiomic):ti,ab,kw OR (radiogenomic):ti,ab,kw OR (radiomics-based):ti,ab,kw OR (radiomic signature):ti,ab,kw | 419 |
| #13 | #7 OR #8 OR #9 OR #10 OR #11 OR #12 | 32188 |
| #14 | MeSH descriptor: [Immune Checkpoint Inhibitors] explode all trees | 75 |
| #15 | (Immune Checkpoint Inhibitors):ti,ab,kw OR (Immunotherapy):ti,ab,kw OR (Checkpoint Inhibitors, Immune):ti,ab,kw OR (Immune Checkpoint Inhibitor):ti,ab,kw OR (Checkpoint Inhibitor, Immune):ti,ab,kw | 12191 |
| #16 | (Immune Checkpoint Blockers):ti,ab,kw OR (Checkpoint Blockers, Immune):ti,ab,kw OR (Immune Checkpoint Blockade):ti,ab,kw OR (Checkpoint Blockade, Immune):ti,ab,kw OR (Immune Checkpoint Inhibition):ti,ab,kw | 578 |
| #17 | (Checkpoint Inhibition, Immune):ti,ab,kw OR (PD-L1 Inhibitors):ti,ab,kw OR (PD L1 Inhibitors):ti,ab,kw OR (PD-L1 Inhibitor):ti,ab,kw OR (PD L1 Inhibitor):ti,ab,kw | 1381 |
| #18 | ('Programmed Death-Ligand 1 Inhibitors'):ti,ab,kw OR ('Programmed Death Ligand 1 Inhibitors'):ti,ab,kw OR ("PD-1-PD-L1 Blockade"):ti,ab,kw OR ("Blockade, PD-1-PD-L1"):ti,ab,kw OR ("PD 1 PD L1 Blockade"):ti,ab,kw | 245 |
| #19 | (CTLA-4 Inhibitors):ti,ab,kw OR (CTLA 4 Inhibitors):ti,ab,kw OR (CTLA-4 Inhibitor):ti,ab,kw OR (CTLA 4 Inhibitor):ti,ab,kw OR (Cytotoxic T-Lymphocyte-Associated Protein 4 Inhibitors):ti,ab,kw | 271 |
| #20 | (Cytotoxic T Lymphocyte Associated Protein 4 Inhibitors):ti,ab,kw OR (Cytotoxic T-Lymphocyte-Associated Protein 4 Inhibitor):ti,ab,kw OR (Cytotoxic T Lymphocyte Associated Protein 4 Inhibitor):ti,ab,kw OR (PD-1 Inhibitors):ti,ab,kw OR (PD 1 Inhibitors):ti,ab,kw | 2947 |
| #21 | (PD-1 Inhibitor):ti,ab,kw OR (Inhibitor, PD-1):ti,ab,kw OR (PD 1 Inhibitor):ti,ab,kw OR (Programmed Cell Death Protein 1 Inhibitor):ti,ab,kw OR (Programmed Cell Death Protein 1 Inhibitors):ti,ab,kw | 4228 |
| #22 | #14 OR #15 OR #16 OR #17 OR #18 OR #19 OR #20 OR #21 | 16998 |
| #23 | #6 AND #13 AND #22 | 75 |

**75 Results**
